# Ductal carcinoma *in situ *of the breast (DCIS) with heterogeneity of nuclear grade: prognostic effects of quantitative nuclear assessment

**DOI:** 10.1186/1471-2407-7-174

**Published:** 2007-09-10

**Authors:** Judith-Anne W Chapman, Naomi A Miller, H Lavina A Lickley, Jin Qian, William A Christens-Barry, Yuejiao Fu, Yan Yuan, David E Axelrod

**Affiliations:** 1 National Cancer Institute of Canada Clinical Trials Group, Queen's University, 10 Stuart Street, Kingston, Ontario K7L 3N6, Canada; 2 Department of Pathology, University Health Network and University of Toronto, Princess Margaret Hospital, 610 University Avenue, Toronto, Ontario M5G 2M9, Canada; 3 Henrietta Banting Breast Centre, Women's College Hospital, University of Toronto, 76 Grenville Street, 7th floor, Toronto, Ontario M5S 1B2, Canada; 4 Department of Statistics and Actuarial Science, Faculty of Mathematics, University of Waterloo, 200 University Avenue West, Waterloo, Ontario N2L 3G1, Canada; 5 Equipoise Imaging LLC, 4009 St. Johns Lane, Ellicott City, MD 21042, USA; 6 Department of Mathematics and Statistics, York University, 4700 Keele Street, Toronto, Ontario M3J 1P3, Canada; 7 Department of Genetics and Cancer Institute of New Jersey, Rutgers University, 604 Allison Road, Piscataway, NJ 08854-8082, USA

## Abstract

**Background:**

Previously, 50% of patients with breast ductal carcinoma *in situ (*DCIS) had more than one nuclear grade, and neither worst nor predominant nuclear grade was significantly associated with development of invasive carcinoma. Here, we used image analysis in addition to histologic evaluation to determine if quantification of nuclear features could provide additional prognostic information and hence impact prognostic assessments.

**Methods:**

Nuclear image features were extracted from about 200 nuclei of each of 80 patients with DCIS who underwent lumpectomy alone, and received no adjuvant systemic therapy. Nuclear images were obtained from 20 representative nuclei per duct, from each of a group of 5 ducts, in two separate fields, for 10 ducts. Reproducibility of image analysis features was determined, as was the ability of features to discriminate between nuclear grades. Patient information was available about clinical factors (age and method of DCIS detection), pathologic factors (DCIS size, nuclear grade, margin size, and amount of parenchymal involvement), and 39 image features (morphology, densitometry, and texture). The prognostic effects of these factors and features on the development of invasive breast cancer were examined with Cox step-wise multivariate regression.

**Results:**

Duplicate measurements were similar for 89.7% to 97.4% of assessed image features. For the pooled assessment with ~200 nuclei per patient, a discriminant function with one densitometric and two texture features was significantly (p < 0.001) associated with nuclear grading, and provided 78.8% correct jackknifed classification of a patient's nuclear grade. In multivariate assessments, image analysis nuclear features had significant prognostic associations (p ≤ 0.05) with the development of invasive breast cancer. Texture (difference entropy, p < 0.001; contrast, p < 0.001; peak transition probability, p = 0.01), densitometry (range density, p = 0.004), and measured margin (p = 0.05) were associated with development of invasive disease for the pooled data across all ducts.

**Conclusion:**

Image analysis provided reproducible assessments of nuclear features which quantitated differences in nuclear grading for patients. Quantitative nuclear image features indicated prognostically significant differences in DCIS, and may contribute additional information to prognostic assessments of which patients are likely to develop invasive disease.

## Background

Ductal carcinoma *in situ *(DCIS) of the breast is being diagnosed more frequently as a result of mammographic screening. Nuclear grade is used as a major determinant of therapy for these patients, although assignment of a grade is a subjective evaluation and there is inconsistent evidence for prognostic importance [1-11].

In our previous study of *in situ *duct carcinoma [12], we identified considerable heterogeneity of grading with about 50% of the patients exhibiting more than one grade (different grades in the same duct, different grades in different ducts in the same area, or different grades in different areas). We attempted to incorporate this heterogeneity by evaluating nuclear grade in terms of worst or predominant grade; however, nuclear grade assessed through either of these methods was not significantly associated with local DCIS recurrence, or the development of invasive disease. There were previously no clinical or histologic factors with significant (p ≤ 0.05) univariate or multivariate associations with the development of invasive carcinoma, although this cohort had experienced an unexpectedly high early development of invasive carcinoma, at median 5 years follow-up, that was predominantly associated with "lumpectomy alone" (referring to local excision alone, rather than the presence of a lump). We hypothesized that the heterogeneity of grading contributed to the lack of apparent clinical significance for nuclear grade. A quantitative assessment of grading could be both more reproducible and provide better prognostic discrimination. Image analysis of nuclei allows a quantitative assessment of this kind.

We present here a detailed clinical study of heterogeneity of DCIS as determined by both histologic evaluation and image analysis. We utilize the same DCIS patient cohort and clinical and pathologic factors as in the previous study by Miller, et al. [12] to provide an estimate of additional benefit from considering quantitative nuclear features determined by image analysis. The focus of this investigation was to examine whether image analysis features impacted prognosis with respect to the development of invasive breast cancer.

## Methods

### Patients

Women's College Hospital began the routine use of mammography in the mid-1960's, establishing a breast imaging expertise. This led to increased detection of more breast DCIS at the Henrietta Banting Breast Cancer Center, a multidisciplinary assessment center for breast diseases, in the study period between 1979 and 1994 [12]. Analysis of patient records from the practices of the group of teaching surgeons identified 260 women who were diagnosed as having DCIS of the breast. Study cases included had (a) DCIS confirmed by pathology review, (b) histology slides of the initial DCIS and most subsequent carcinomas available for review, (c) no previous breast or other malignancy, and (d) a detailed follow-up to 1997 [12]. One hundred and eighteen patients were excluded for the following reasons: 18 on review did not have DCIS; 100 had previous carcinoma, and 18 had no (or limited) follow-up or primary histologic slides were not available for review. The data for the remaining 124 patients with DCIS formed the basis of the previous study. These patients had a median 5.0 years of follow-up. The focus was the 88 patients who underwent lumpectomy alone since this group experienced most of the subsequent clinical events: 17 of 19 recurrences of DCIS, with the other 2 observed in 18 patients who underwent lumpectomy followed by adjuvant radiotherapy, and all 19 DCIS recurrences were ipsilateral at median interval 2.6 years, range 5.3 months to 5.7 years; 11 of 12 developments of invasive carcinoma, the eleven invasive cancers were 6 ipsilateral and 5 contralateral at median 1.8 years, range 9.5 months to 6.6 years. The median time for development of ipsilateral invasive carcinoma was 1.6 years, range 9.5 months to 6.5 years. The twelfth diagnosis of invasive carcinoma in the original DCIS series was an ipsilateral axillary lymph node, which occurred in a patient who was initially treated with simple mastectomy [13] and who is excluded from these investigations. Seventy-eight of the 88 patients (88%) were detected mammographically. None of the 88 patients received adjuvant radiotherapy or systemic therapy.

The specimens were processed uniformly in a manner consistent with standards at the time of the biopsies [12]. Specimens were fixed in 10% neutral buffered formalin. Tissue blocks were created with uniform section thickness of 3–4 microns. Tissue evaluated had not been examined at previous frozen section. For mammographically detected lesions, tissue was sampled rather than assessed in toto, with sampling directed to the area marked by dye instilled preoperatively and/or area marked by a needle placed intraoperatively by a radiologist. Sampling of other specimens was directed by the gross appearance of the specimen. Several assessments were made to reflect size: (a) estimate from the gross description, (b) maximum dimension per slide, and (c) number of slides with DCIS involvement.

The percent parenchyma involved (<10%, 10 to 50%, >50%) was assessed to reflect the proportion of the total parenchyma (stroma and all ducts and lobules) in the areas on the slides containing DCIS that was occupied by the involved ducts. Percentage parenchyma was based on both fibrous and fatty stroma, and was determined on sections which contained DCIS. This reflected whether the involved ducts were concentrated within the parenchyma (higher percentage) or more diffusely scattered (lower percentage). Duct distribution tended to be uniform, and a single categorization could be assigned to each patient.

Results were obtained by considering the maximum size of the DCIS on any slide and the percentage of the parenchyma involved.

Resection margins had been painted with silver nitrate. The shortest distance between an involved duct and resection margin was measured microscopically with an ocular micrometer (in millimeters). The presence of an uninvolved duct between DCIS and painted margin (reported as cannot assess, not present, present) was also assessed.

The DCIS was classified into architectural patterns: solid, cribriform, micropapillary, other (e.g., papillary, apocrine, clinging). All of the types present, the predominant (most extensive) type present, and the type with least architectural differentiation (solid versus all other) were recorded. We used the results obtained by considering predominant architecture and least architectural differentiation: from previous paper [12], 51% of tumours were solid by worst architecture, while 33% were solid by predominant architecture.

Calcifications included present (amorphous or crystalline) or not present. Necrosis was central confluent (comedo) versus not: 67% of patients had necrosis[12].

DCIS was graded as 1, 2, and 3 (by the Van Nuys classification scheme [14]. When more than one grade was present, the worst (highest) grade was recorded, as well as all grades present and the predominant (most extensive) grade.

### Image analysis

Good quality computer images for the purpose of image analysis could be obtained from the archived hematoxylin and eosin (H&E) stained slides of 80 of these patients; 3 patients did not have slides available for assessment, while 5 had H&E stained slides which resulted in poor quality images that were unsuitable for further evaluation. These 80 patients are the subset considered for the current study. Digital images of the slides of the 80 study patients were acquired under the supervision of a breast pathologist (NM).

Representative H&E stained slides for each patient were selected that demonstrated the nuclear grading previously observed for that patient. A maximum of 2 slides per patient biopsy were selected. Two fields were selected so that ducts were sufficiently concentrated to contain a minimum of 5 ducts per field in which the nuclear grading was represented (either two fields in one slide, or one field on each of 2 slides). Affected duct spaces were contiguous. A low, 10 times, magnification was used for identification of appropriate sampling regions, while 40 times magnification was used for capturing images. A computer image was acquired for each of 5 ducts in one field and this was repeated for 5 ducts in the other field. Image features were measured for each of approximately 20 representative nuclei per duct, for a total of about 200 nuclei for each patient. Thirty-nine computer image features were extracted for each nucleus that described morphometry (size and shape of nuclei), densitometry (amount of staining of the nuclei), and texture (arrangement of staining in the nucleus). Images were acquired using NIH-Image software v.1.57 written by Wayne Rasband [15]. Nuclear morphologic and densitometric features were measured with NIH-Image v.1.62b34-Arnv software. Texture features were measured using TextureCalc v.1.1ax software written by W.C.-C. All nuclear images were segmented by a single person (DA). Some image feature calculations and the merging of all nuclear image feature data to per duct, per field and per person attribution were accomplished with StatView v 5.01 (Brain Power, Calabasas, CA) software. Nuclear images were segmented for image analysis without knowledge of the corresponding clinical features or pathologist's grading. Nuclei distributed throughout the image field were segmented in order to assure a representative sample. Nuclear images that were incomplete (cut off at the edges of the field) or overlapping were not included. The few image fields that were indistinct or out of focus were not used.

### Image analysis features

For each nucleus, 39 features were determined in three categories. (i) Morphometry: area, perimeter, ellipse major axis, ellipse minor axis, ellipticity (major axis/minor axis), shape form factor (4 × pi × area/perimeter squared), and roundness b (4 × area/pi × ellipticity squared) [16]. (ii) Densitometry: mean density, standard deviation of density, modal density, minimum density, maximum density, sum density (mean density × area, used instead of I.O.D. of NIH-Image), range density. (iii) Markovian texture features [17,18] were calculated from the Markovian co-occurrence matrix of pixel densities with a step size of 2. They were angular second moment, contrast, correlation, variance, inverse difference moment, sum average, sum variance (corrected from [18]), difference average, difference variance, initial entropy, final entropy, entropy, sum entropy, difference entropy, coefficient of variation, peak transition probability, diagonal variance, diagonal moment, second diagonal moment, product moment, and triangular symmetry. Additional texture features, calculated from the binned histogram of pixel gray scale values, included histogram mean, histogram variance, histogram skewness, and histogram kurtosis. The mathematical formulae defining these image features can be found in the cited references.

### Prognostic factors

The clinical factors recorded on these patients were age (in years) and type of presentation (mammographic, clinically palpable, bloody nipple discharge). The histologic factors previously evaluated [12] were maximum DCIS size (cm), percentage of parenchyma involved with DCIS (<10%, 10–50%, >50%), predominant architecture (0 – cribriform/micropapillary/other, 1 – solid), worst architecture (0 – cribriform/micropapillary/other, 1 – solid), nuclear grade [by the Van Nuys Classification system worst (nuclear grade 1, 2, 3); also, predominant (nuclear grade 1, 2, 3)], necrosis [none, confluent (comedo-like)], calcification (none, crystalline/amorphous), measured margin (zero margin, <1 mm, 1–5 mm, >5 mm), presence of uninvolved intervening duct (not assessable, no, yes), Van Nuys Prognostic Index. In addition, 39 nuclear image features were determined for about 200 nuclei per patient.

For each patient, the image data were pooled across i) all nuclei in a duct (10 assessments), ii) all nuclei in a field (2 assessments), and iii) all nuclei for a patient (1 assessment) to yield a summary feature value [adjusted mean = mean/(standard error of the mean)], for each of the 39 image features for nuclei of the 13 different assessments per patient: 10 ducts, 2 fields, 1 overall. In addition, grading discriminant classification functions, that are weighted combinations of image features, described below in the Analysis section, were assessed as prognostic factors.

Thirteen different assessments, corresponding to the 13 different ways of pooling the image analysis feature data, were performed to examine the effects of DCIS heterogeneity on apparent associations with clinical outcome. In other contexts, investigations have been restricted to single ducts, fields, or pooled per person assessments without an examination of replicability.

### Events

A new diagnosis of breast carcinoma made more than 90 days after the initial surgery was designated as an event. Invasive carcinoma in this group of patients occurred about equally in both the ipsilateral and contralateral breast which is consistent with the findings of some others [19-21], including those for potentially lower risk DCIS patients [21]. Using t-tests, there was no statistically significant difference in image analysis features between patients who developed invasive disease ipsilaterally, as opposed to contralaterally. For these investigations, an event was considered to be development of invasive carcinoma whether ipsilateral or contralateral. There were no deaths from breast cancer, or another cause, in this group of patients over the study period.

### Analyses

Statistical analyses were performed with BMDP PC Dynamic Version 7.0 (same as BMDP-XP, Statistical Solutions, Sagua, MA).

Image analysis pre-processing of data included for each image feature and each patient, 1) Levene's tests for equality of variance between ducts and fields for each person and between people, 2) the use of the mean/S.E.M. of image features on a duct, field and patient basis, resulting from indications in Levene's tests of significant evidence against assumption of equal variances, 3) per duct, per field, and per person grading disciminant classification functions from forward step-wise Fisher linear discriminant analyses, using an entry p-value of p ≤ 0.05, and 4) assessment of the correct classification by the grading discriminant classification functions using jackknifed (leave-one-out) classification of patients. Standardized coefficients for canonical variables in the discriminant function are reported.

The histologic, clinical, and image analysis factors were assessed with respect to whether they were associated with the development of invasive disease. Univariate assessments were with Kaplan-Meier plots and the Wilcoxon (Peto-Prentice) test statistic. For each image feature, standard image analysis cut-points at the means of the data were utilized after confirmation that the data were approximately symmetric.

Multivariate assessments were with Cox forward step-wise regressions, using the likelihood ratio criterion (~χ^2^_(1)_, p ≤ 0.05) as the test statistic to determine if a factor would be added to the model. Since we had no knowledge of which of the image analysis features assessed would best reflect a patient's DCIS, or the extent to which differences in image features might relate to prognoses, we performed 13 sets of multivariate analyses, corresponding to the 13 generations of image feature factors per patient: per 10 ducts, 2 fields, 1 pooled across 2 fields.

This study was approved by the Ethics Review Board at Women's College Hospital, Toronto, Ontario, Canada.

## Results

### Patients

Archival H&E slides were satisfactory for image analysis for the majority of this DCIS cohort. Only 5.7% (5/88) patients had slides that were of too poor quality; three patients did not have slides available for assessment. Table 1 summarizes the heterogeneity of nuclear grade assessed in the 80 patients for whom image analysis was performed. In total, thirty-nine of the 80 (49%) patients had more than one nuclear grade on histologic evaluation. For analysis purposes, the patients were grouped two ways by nuclear grading type: the focus of reporting by Van Nuys classification of worst grade, except the single patient with nuclear grade 1 was grouped with grade 2; and, Group A (grade 1 only, grades 1 and 2, or grade 2 only), Group B (grades 1, 2, and 3, or grades 2 and 3), and Group C (grade 3 only). This latter grouping of patients by grade was used to reflect the heterogeneity observed. Nuclear grade 1 could not be studied on its own since only one patient had grade 1 only.

**Table 1:** Number of patients by pathologic grading type

		Group	
		A	B

Van Nuys Grade 1		1	
Grade 1 only	1		
Van Nuys Grade 2		31	
Grades 1 and 2	8		
Grade 2 only	23		
Van Nuys Grade 3			48
Grades 2 and 3	29		
Grades 1, 2, 3	2		
Grade 3 only	17		
Total by group		32	48

### Image analysis

All nuclear regions of interest were segmented manually by the same operator (DA). Reproducibility of manual segmentation was determined by repeatedly segmenting the same nucleus (CV = 3.4%, n = 150). In order to determine the reproducibility of extracted feature values, independent measurements were made of the same nucleus in images captured at different times. Ten or more nuclei, identified from images of specimens of seven patients, were segmented at two different times without knowledge of the previously segmented region of interest, or of the extracted feature values. The differences between the pairs of measurements of the same nucleus were determined with a two-tailed t-test. (The null hypothesis was that the differences were equal to zero, at the 0.05 level of significance.) The percent of feature values that were not statistically different in duplicate measurements ranged from 89.7% to 97.4% among nuclei of the seven patients. Further, there were no statistically significant differences for 23 of the 39 feature values in 7/7 pairs of images, and 37 of 39 feature values in at least 5/7 pairs of images.

### Classification of patients by image analysis

Patients were classified into two groups as shown in Table 1. These grading groups were used in the discriminant analyses for nuclei in field 1 (80 patients), field 2 (79 patients), and overall pooled across both fields (80 patients). In each instance (field 1, field 2, and overall across both fields), there were image features significantly associated with the grading classifications, p < 0.001 for each. The discriminant function for the first field included two morphological features reflecting the size of nuclei (perimeter and shape-form-factor) and three texture features reflecting the arrangement of DNA in the nucleus (sum entropy, product moment, difference average). Discriminant analysis of the second field included one morphological feature (perimeter) and one texture feature (angular second moment). The pooled analyses across both fields indicated one densitometric feature (sum density) and two texture features (diagonal moment and product moment). Different image analysis features were obtained for the first field, second field, and both fields. Correct jackknifed (leave-one-out) classification of the nuclear grading with the image features was respectively, 83.8%, 81.0% and 78.8%.

**Figure 1:**
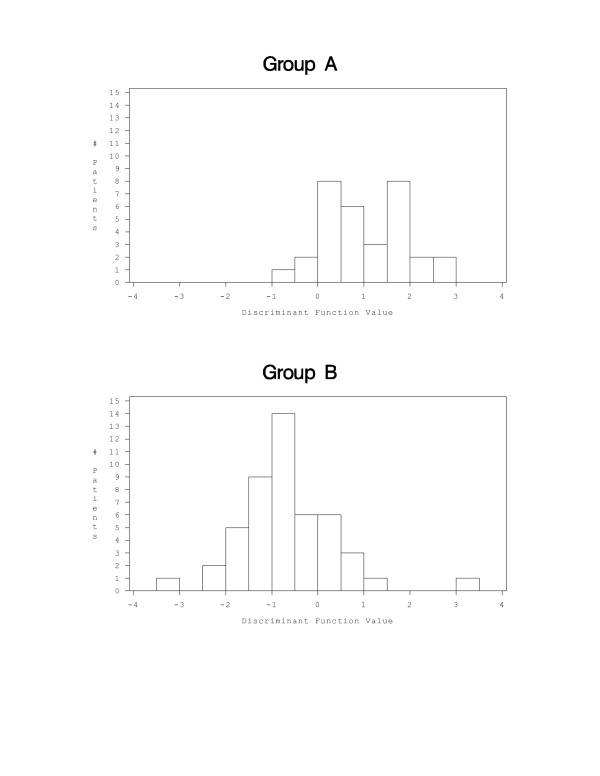
Distribution of patients between the grading groups given in Table 1.: A. Group A, B. Group B. The value of the discriminant function for each patient is determined by a weighted combination of image features significantly associated (p < 0.001) with the characteristics of the grading groups, factors that dealt with sum of pixel intensities (sum density) and changes in spatial arrangement (diagonal moment and product moment).

Discrimination using both fields would be most representative for a patient. There was significant separation (p < 0.001) by grade with factors that dealt with sum of pixel intensities (sum density) and changes in spatial arrangement (diagonal moment and product moment). The effects of the discriminant classification function [0.9899 × (sum density) - 0.658 × (diagonal moment) - 0.43424 × (product moment) + 2.53292] are shown visually in Figure 1, with the distribution of patient values within each of the two nuclear grading groups. Although the discriminant function was optimized to provide the best classification of patients within the two grading groups, there was considerable overlap between patients in the two grading groups. Table 2 indicates the accuracy of image analysis classification by nuclear grading group. Image analysis features correctly classified 75.0% of patients in Group A and 81.2% in Group B.

**Table 2:** Discriminant function classification of patients by grading group

Group	Patients in group	Number of Patients classified into group		Percent correct
		A	B	
A	32	24	8	75.0
B	48	9	39	81.2
Total	80	33	47	78.8

### Prognostic factors

No clinical or pathologic factors were previously associated with the development of invasive disease [12]. The addition of image analysis factors led to the associations listed in Table 3. Image analysis derived features predominate the significant factors indicated by multivariate analyses although the particular factors differ among the 13 sets of assessments (10 ducts, 2 fields, and 1 overall pooling per patient). Differences were also indicated between pooled data for the two fields (~100 nuclei/patient) and the overall pooling across both fields (~200 nuclei). The texture feature 'difference entropy' was the only factor with a demonstratively consistent indication, being included in 10 of 13 assessments and all 3 pooled models across fields. The lack of stability in results suggested it would be premature on the basis of these data to draw conclusions about features, although there was definite evidence to support the prognostic relevance of quantitative image features. Nuclear grade was only significant in the fourth assessment in field 1. The discriminant classification function was significantly associated with the development of invasive disease (p < 0.001) only for field 2. The partitioning of patients into three groups by heterogeneity of grading similarly yielded 13 sets of factors; however, the resulting discriminant classification function with minor ellipse axis and peak transition probability was significantly associated with the development of invasive disease in both the field 2 and overall pooled assessments.

**Table 3:** Clinical, histologic, and image analysis factors affecting development of invasive disease by image analysis assessment

**Clinical, histologic, and image analysis factors by assessment**			
Field 1		Field 2	

Factors^a^	P-value	Factors^a^	P-value

Assessment 1		Assessment 6	
Densitometry (Minimum density)	0.001	Texture (Difference entropy)	<0.001
Texture (Histogram mean)	0.02	Morphometry (Ellipse major axis)	<0.001
		Texture (Angular second moment)	0.001

Assessment 2		Assessment 7	
Texture (Difference entropy)	0.01	Densitometry (Mean density)	0.001
Morphometry (Ellipse minor axis)	0.03	Texture (Difference entropy)	0.001
		Texture (Diagonal moment)	0.04

Assessment 3		Assessment 8	
Texture (Difference entropy)	0.01	Densitometry (Ellipse minor axis)	0.01
		Densitometry (Maximum density)	0.003
		Morphometry (Shape form factor)	0.04

Assessment 4		Assessment 9	
Texture (Sum variance)	0.003	Texture (Difference entropy)	<0.001
Texture (Difference entropy)	<0.001	Texture (Contrast)	<0.001
Texture (Second diagonal moment)	<0.001	Morphometry (Ellipse major axis)	0.001
Nuclear grade	0.001	Age	0.02
Morphometry (Ellipse major axis)	0.01	Densitometry (Range density)	0.03

Assessment 5		10	
Densitometry (Minimum density)	<0.001	Morphometry (Shape form factor)	0.001
Measured margin	<0.001	Texture (Histogram mean)	0.02
Texture (Variance)	<0.001	Texture (Difference entropy)	0.01
Texture (Histogram Mean)	<0.001	Measured margin	0.05
Texture (Contrast)	0.002		

Field 1 Overall		Field 2 Overall	
Assessment 11		Assessment 12	

Densitometry (Minimum density)	0.005	Texture (Difference entropy)	<0.001
Texture (Difference entropy)	0.01	Discriminant classification function	<0.001
Texture (Diagonal moment)	0.04	Parenchymal involvement	0.001
			
Both Fields Overall – Assessment 13			
Texture (Difference entropy)			p < 0.001
Texture (Contrast)			p < 0.001
Texture (Peak transition probability)			p = 0.01
Densitometry (Range density)			p = 0.004
Measured margin			p = 0.05

^a ^Factors significantly (p ≤ 0.05) associated with development of invasive disease, in the order entered into the step-wise forward Cox regression models.

**Figure 2:**
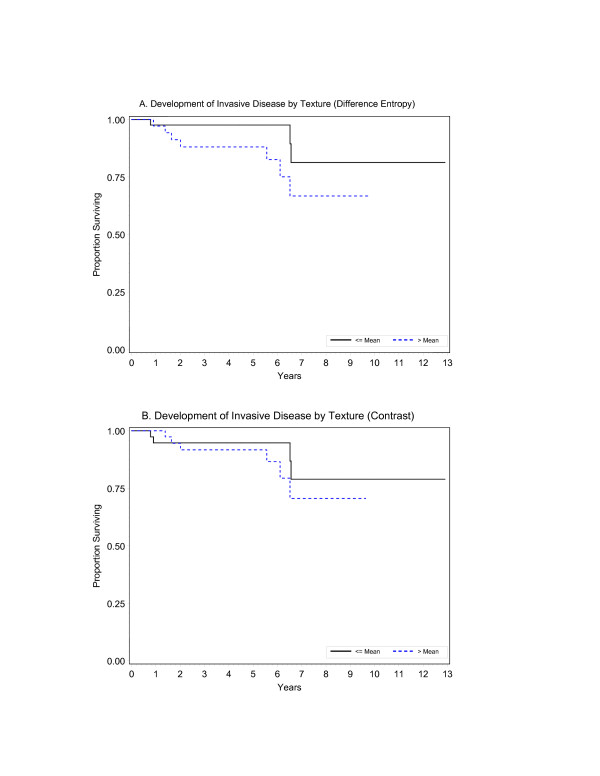
Kaplan-Meier plots for the two image analysis factors significantly (p < 0.001) associated with development of invasive disease in the multivariate assessments: A. Texture (difference entropy) (p < 0.001). B. Texture (contrast) (p < 0.001).

The factors indicated in the multivariate analyses of the overall pooled data represent the best available summary for the patients. The factors significantly associated with the development of invasive disease were three texture features quantifying the degree of order (difference entropy p < 0.001), contrast (p < 0.001), and strongest spatial relationship between pixels (peak transition probability, p = 0.01), a densitometry feature (range in pixel intensity, range density, p = 0.004), and measured margin [larger values associated with increased risk of developing invasive breast cancer (p = 0.05)]. Figure 2 shows the univariate Kaplan-Meier plots for the first two factors associated with development of invasive disease.

## Discussion

Nuclear grading is an important component of medical treatment decisions for DCIS. The determination of clinical relevance for any system of assigning a nuclear grade is hampered by the long follow-up time required to assess the sequelae of DCIS recurrence, or the more serious development of invasive breast cancer. Several different guidelines have been proposed in the literature for assignment of nuclear grade [e.g. [2,11]], however none of these allow incorporation of heterogeneity of grade which has been observed by others and by us [e.g. [11,12,22]], or mixed architecture [22]. Using the Van Nuys grading system [2,14] of worst grade, we previously found that grade was not associated with either DCIS recurrence or development of invasive disease [12]. Since 50% of our cohort of DCIS patients exhibited more than one nuclear grade, we previously attempted to evaluate heterogeneity of grading by evaluating the significance of predominant nuclear grade. However, we did not find that predominant grade was associated with clinical outcome. Further, nuclear grade was not significantly associated with outcome in the much larger series of NSABP-17 at median 8 year follow-up [6].

Description of nuclei on a continuous scale might be preferable to classification into three discrete grades because it would allow finer differentiation of patients' risk [12]. As well, a more objective system of assigning a grade would be preferable [23]. We therefore evaluated our patient cohort using image analysis to measure multiple features of many individual nuclei, and characterized heterogeneity within the DCIS lesion of each patient in a study designed to reveal differences between different ducts and different fields. The focus here was on the potentially serious sequelae of development of invasive disease in a cohort that experienced an unusually high rate of this after only a median 5 year follow-up. Although this early detection may in part be attributable to lack of detection of pre-existing invasive disease, the primary assessment was by breast disease experts with regional standards of care for the time period of patient accrual. As well, the predominance of events occurred in patients receiving lumpectomy alone, who a priori did not have as advanced DCIS; patients were more likely to be managed with adjuvant radiotherapy following lumpectomy, or with mastectomy, if the DCIS was larger than 1 cm (p = 0.01), grade 3 (p = 0.03 by worst grade, p = 0.01 by predominant grade), or had confluent necrosis (p = 0.01) [12]. Only 7 of the full DCIS cohort had treatment directed by participation in NSABP-17 or NSABP-24. The prognostic associations of image analysis features with early development of invasive disease is important, given the previous lack of associations with our clinical or pathologic factors.

The image features had demonstrative prognostic value, although there were differences in the specific set of features for each assessment (ducts, fields, and overall). At least three conditions may have contributed to this inconsistency, numbers of nuclei, correlation between features, and biologic heterogeneity between ducts/fields. First, there were differences in the number of nuclei in each assessment: Assessments 1 to 10 included 20 nuclei per patient, whereas Assessments 11 and 12 had 100 nuclei each, and Assessment 13 had 200 nuclei. Assessment 13, with the largest number of nuclei, should provide most representative grading on a per person basis; however, some as yet unestablished nuclear characteristics in a heterogeneous DCIS may differentially affect prognosis. A second condition that may have contributed to differences is the correlation between image features, especially those in the same category. For instance, morphological features, ellipse major axis, ellipse minor axis, and measures of nuclear size should be correlated unless there is large variation in the shape of various nuclei of the same patient. However, image analysis is still an investigational tool, so consideration of clinical relevance for particular factors is exploratory, and hypothesis generating. Thus, correlated features, or groups of features, listed separately for different assessments may have similar prognostic value. Thirdly, the existence of heterogeneity between nuclei within the same patient, indicated by mixed nuclear grades, would be expected to influence the selection of image features in different fields of the same patient. In this last context, differences in selection of image features confirms the ability of image analysis to detect and quantify that heterogeneity. The heterogeneity between ducts of the same patient that was revealed in this study provides a cautionary note for interpretation of data from microdissection or microarray methodologies in which a small number of samples each with only a few cells are used to characterize a tumor, since the heterogeneity of a tumor in vivo may not be reflected in such small samples [24,25].

Finally, we cannot eliminate the possibility that a more thorough examination of the DCIS would have detected the presence of constitutive ipsilateral invasive disease, although ipsilateral invasive disease could as well be related to multiple foci of tumour which cannot be perceived at the time of primary therapy [6]. It is important to note that the association of larger measured margins with increased development of invasive disease occurred in a clinical setting where surgeons may have attempted wider excisions in patients perceived to have more extensive disease, and hence patients were potentially more likely to develop invasive breast cancer [12]. A recent prospective study of patients with predominant grade 1 or 2, mammographic extent less than or equal to 2.5 cm treated with wide excision alone and either having final margins ≥ 1 cm, or a re-excision without residual DCIS, and who were not permitted adjuvant Tamoxifen had to close accrual due to meeting predetermined stopping rules for number of local recurrences [21]. Four of these patients had ipsilateral invasive disease and four of the five who developed contralateral invasive disease had this invasive disease as their first failure; the invasive local recurrence rate was 2.5%, 36% of our ipsilateral rate at a similar time interval. It is important to note that like our study, the Wong, et al study [21] did not have complete sequential tissue processing [26].

Image analysis was used to precisely quantitate nuclear morphometric, densitometric, and texture features. It is noteworthy that the addition of the continuous image feature data resulted in finding factors significantly associated with the development of invasive disease [10], where there had been none in previous work [12].

Multivariate analysis using image measurements indicated a multiplicity of factors, rather than a unique set of factors, that were associated with outcome(s). Although the texture feature 'difference entropy' was included in 10 of 13 assessments and all three pooled over the fields, the thirteen assessments led to thirteen distinct sets of predominantly image feature factors significantly associated with the development of invasive breast cancer. Similarly, there were another 13 sets of factors associated with DCIS recurrence. Two conditions may have contributed to this, the heterogeneity within the DCIS lesions and the precision of the image measurements that allowed detection of the heterogeneity. The multiplicity of results represents an ability of image analysis to quantitatively detect finer nuclear heterogeneity than was qualitatively identified in histologic evaluation [12].

Morphometric features of nuclear perimeter and nuclear area of breast DCIS were found to be associated with grade, necrosis, polarization, comedo architecture, and tumor size, although their relationship to the development of invasive disease was not reported [22]. Image analysis of nuclear features has been found to provide quantitative information that can contribute to diagnosis and can have prognostic value for carcinomas of the breast [27-36] and other sites [prostate, [37]; colorectal mucosa, [38]]. The assessments here were specific to nuclei in DCIS lesions and did not include cytoplasm or stroma. This was possible because images of nuclei were manually segmented. When regions outside of the nuclei were included in the extraction of image feature values, there were no features that were significantly associated with DCIS recurrence or progression to invasive disease. These results emphasize that differences detected by image analysis that are specifically in nuclei may be informative.

Our results must be viewed as hypothesis generating. Larger patient populations would be desirable for confirmatory purposes, and delineation of relevance for particular image analysis features. However, as there is currently no well-defined low-risk subgroup of patients who can reasonably forego adjuvant therapy [21,39], this should be a worthwhile exploration.

## Conclusion

Our results suggest that it would be worthwhile to investigate tumours for other cohorts where there is heterogeneity of nuclear grade. The images acquired in this study were from archived biopsy specimens stained with hematoxylin and eosin (H&E). This suggests that other DCIS cohorts, for which there are stored slides stained with H&E and for which there is long term follow-up information, could be used to yield valuable information.

The heterogeneity between ducts that was revealed in this study provides a cautionary note for interpretation of data from microdissection or microarray methodologies in which a small number of samples with only a few cells are used to characterize a tumor, since the heterogeneity of a tumor *in vivo *may not be reflected in such small samples [24,25].

The prognosis of patients with breast ductal carcinoma in situ (DCIS) to develop invasive carcinoma can be improved by adding quantitative nuclear features obtained by image analysis to clinical factors and histopathological grades. Image analysis may be especially useful in assessing patients with mixed nuclear grades.

## Competing interests

WC-B is President and Chief Scientist at Equipose Imaging LLC. The other authors have no competing interests.

## Authors' contributions

All authors read and approved the final manuscript. DA acquired the data, was involved in design of the study, analysis and interpretation of results, drafting and preparing the final manuscript. JC was involved in design of the study, analysis and interpretation of results, drafting and preparing the final manuscript. NM was involved in design of the study, pathological review of specimens, interpretation of results, and preparing the final manuscript. WC-B wrote the software used to extract the image features and reviewed the manuscript. JQ, YY and YF were involved in analysis of data and reviewed the manuscript. HL was involved in acquisition of specimens through surgical management of patients and reviewed the manuscript.

## Pre-publication history

The pre-publication history for this paper can be accessed here:


